# Workshop-based learning and networking: a scalable model for research capacity strengthening in low- and middle-income countries

**DOI:** 10.1080/16549716.2022.2062175

**Published:** 2022-06-22

**Authors:** Celine Perier, Emmanuel Nasinghe, Isabelle Charles, Leoson Junior Ssetaba, Vida Ahyong, Derek Bangs, P. Robert Beatty, Nadine Czudnochowski, Amy Diallo, Eli Dugan, Jacqueline M. Fabius, Hildy Fong Baker, Jackson Gardner, Stephen Isaacs, Birungi Joanah, Katrina Kalantar, David Kateete, Matt Knight, Maria Krasilnikov, Nevan J. Krogan, Chaz Langelier, Eric Lee, Lucy M. Li, Daniel Licht, Katie Lien, Zilose Lyons, Gerald Mboowa, Ivan Mwebaza, Savannah Mwesigwa, Geraldine Nalwadda, Robert Nichols, Maria Elena Penaranda, Sarah Petnic, Maira Phelps, Stephen J. Popper, Michael Rape, Arthur Reingold, Richard Robbins, Oren S. Rosenberg, David F. Savage, Samuel Schildhauer, Matthew L. Settles, Ivan Sserwadda, Sarah Stanley, Cristina M. Tato, Alexandra Tsitsiklis, Erik Van Dis, Manu Vanaerschot, Joanna Vinden, Jeffery S. Cox, Moses L. Joloba, Julia Schaletzky

**Affiliations:** a H. Wheeler Center for Emerging & Neglected Diseases (CEND), University of California, Berkeley, CA, USA; b School of Biomedical Sciences, Makerere University, Kampala, Uganda; c Chan Zuckerberg Biohub, San Francisco, CA, USA; d Department of Molecular and Cell Biology, University of California, Berkeley, CA, USA; e Department of Medicine, University of California, San Francisco, CA, USA; f Quantitative Biosciences Institute (QBI), University of California, San Francisco, CA, USA; g School of Public Health, Center for Global Public Health (CGPH), University of California, Berkeley, CA, USA; h Former CEO, Aduro BioTech, Berkeley, CA, USA; i Department of Plant and Microbial Biology, University of California, Berkeley, CA, USA; j Department of Molecular Biology and Microbiology, Tufts Graduate School of Biomedical Sciences, Boston, MA, USA; k Gladstone Institute of Data Science and Biotechnology, J. David Gladstone Institutes, San Francisco, CA, USA; l Graduate Group in Infectious Diseases and Immunity, School of Public Health, University of California, Berkeley, CA, USA; m Pritzker School of Medicine, University of Chicago, Chicago, IL, USA; n California China Climate Institute, University of California, Berkeley, CA, USA; o Sustainable Sciences Institute, San Francisco, CA, USA; p Quality and Clinical Excellence Department, Providence Queen of the Valley Medical Center, Napa, CA, USA; q School of Public Health, Department of Infectious Disease and Vaccinology, University of California, Berkeley, CA, USA; r Howard Hughes Medical Institute, University of California, Berkeley, CA, USA; s Division of Epidemiology and Biostatistics, School of Public Health, University of California, Berkeley, CA, USA; t Wareham Development, San Rafael, CA, USA; u California Department of Public Health, Richmond, CA, USA; v UC Davis Genome Center, Davis, CA, USA; w Division of Infectious Disease and Vaccinology, School of Public Health, University of California, Berkeley, CA, USA; x Department of Immunology, University of Washington School of Medicine, Seattle, WA, USA; y Division of Infectious Diseases and Immunity, School of Public Health, University of California, Berkeley, CA, USA

**Keywords:** Capacity strengthening, Africa, Uganda, research, infectious diseases

## Abstract

Science education and research have the potential to drive profound change in low- and middle-income countries (LMICs) through encouraging innovation, attracting industry, and creating job opportunities. However, in LMICs, research capacity is often limited, and acquisition of funding and access to state-of-the-art technologies is challenging. The Alliance for Global Health and Science (the Alliance) was founded as a partnership between the University of California, Berkeley (USA) and Makerere University (Uganda), with the goal of strengthening Makerere University’s capacity for bioscience research. The flagship program of the Alliance partnership is the MU/UCB Biosciences Training Program, an in-country, hands-on workshop model that trains a large number of students from Makerere University in infectious disease and molecular biology research. This approach nucleates training of larger and more diverse groups of students, development of mentoring and bi-directional research partnerships, and support of the local economy. Here, we describe the project, its conception, implementation, challenges, and outcomes of bioscience research workshops. We aim to provide a blueprint for workshop implementation, and create a valuable resource for bioscience research capacity strengthening in LMICs.

## Introduction

Strengthening research capacity in low- and middle-income countries (LMICs) is essential to the generation of robust, innovative and locally relevant scientific data. Basic science could have a key role to play in global health research [1,2]. In the last decade, the international call for developing research capacity in sub-Saharan Africa has grown [3–5], and opportunities to support individuals pursuing academic studies and fellowships at academic institutions have increased [6,7]. As reviewed in [8], numerous health research capacity strengthening interventions have been employed in LMICs ranging from simple training programs at individual level to institutional and societal interventions [9]. Overall, development actors now agree that strengthening research capacity encompasses a variety of activities, including trainings to support individuals to acquire research, creating research partnerships/networks, and providing individual support and mentorship [10,11]. It was shown that workshops can play an important role to equip students with cutting edge technologies [12]. Moreover, by ensuring a proper flow of knowledge, workshops generate active participation, boosting the skills and expertise of students.

Makerere University (MU) in Uganda, has multiple long-term, ongoing collaborations with organizations from the USA and Europe, such as the Wellcome Trust, the Swedish International Development Cooperation Agency, the Karolinska Institutet, and the Bill and Melinda Gates Foundation. Though the Alliance for Global Health and Science (The Alliance), UC Berkeley developed a close relationship with MU, implementing innovative workshops as a welcomed step towards modern education with potential benefits to LMIC students.

Following a needs assessment in 2016, the Alliance, an interdisciplinary partnership between the Center of Emerging and Neglected Diseases (CEND), the Biological Sciences Division of the College of Letters and Sciences, and the School of Public Health at UC Berkeley (UCB), developed an in-country workshop curriculum taught by leading US researchers, training Ugandan scientists at host institutions. This article highlights a partnership aiming to draw upon the expertise and strengths of US and Ugandan faculty to enhance scientific training for MU students. Herein, we describe the implementation, the challenges, evaluation and outcomes of bioscience research workshops. The main objectives were to address the following questions: (1) Does an intensive educational workshop increase acquisition of knowledge in a particular field? (2) How does a hands-on, experiential approach encourage the students to explore new areas relevant to the topic and boost their confidence, performance, and productivity? and, (3) Does an in-country 2 weeks Biosciences Training Program expand participants’ networks?

## Program implementation

### Program overview

**Figure 1.**
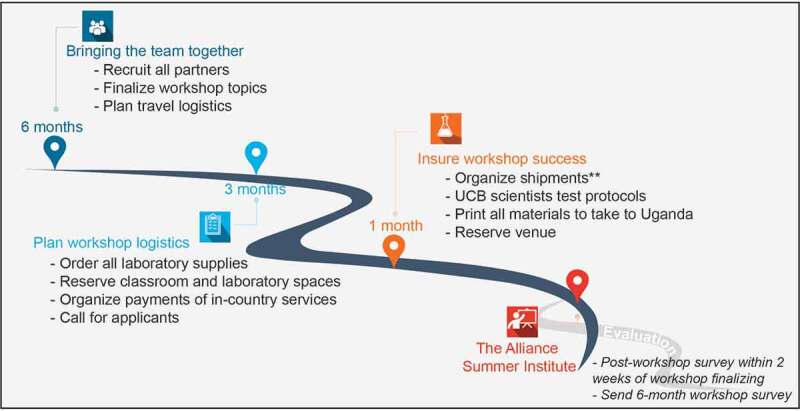
*Roadmap of the MU/UCB biosciences training program implementation*. Involvement of local staff, students and faculty in every phase of the Biosciences Training Program, from planning to follow-up. **All items are repackaged on pallets and shipped to Uganda using World Courier – one shipment for room temperature supplies and one refrigerated shipment. Close coordination with MU receiving department ensures that customs are efficiently cleared and no reagents are lost to insufficient cooling. Average shipment times are 5 to 7 days, and a buffer period between receipt of reagent shipment of one month has to be built in to be able to send another shipment if reagents are lost due to issues with the cold chain.

The Alliance, made possible with generous support from private donors, hosted its first MU/UCB Biosciences Training Program workshop at MU in 2017, emphasizing training in molecular biology, molecular and field epidemiology, critical thinking and grant writing. Since then, the annual MU/UCB Biosciences Training Program offers 5–6 workshops, including experimental lab, non-experimental lab, and professional skills development workshops. The core program of the MU/UCB Biosciences Training Program is pre-determinate, with involvement of the US-faculty each year to ensure continuity, and is re-assessed each year, based on expressed needs and instructor interest. This allows to integrate new technology training, like the CRISPR workshop added in 2019. Each year, the training program is implemented over 6–8 months via conference calls between UCB-based faculty and MU-based faculty (see Figure 1 for more details on the implementation). Faculty develop curriculum and course booklets, and provide a list of reagents, supplies and small equipment needed. Cultivating a strong partnership with the host institution is critical for effective implementation. Participation at all levels of the partner organization, from in-country researchers to staff, ensure that the capacity-strengthening effort is truly owned by the local organization [13,14]. For this reason, a host institution contact and local director of the program needs to be identified early in the program. In our case, a MU faculty member volunteered and has remained a strong and reliable partner through the years, channeling communication from MU, coordinating efforts, logistics, ensuring all efforts and interactions are culturally appropriate, and expectations are met on both sides. The local program director also identifies new contacts and helps to broaden the Biosciences Training Program in response to local needs, and is able to function as a connecting node between Ugandan and US researchers. The local program director is involved in all decisions, enabling shared ownership of the program.

**Figure 2.**
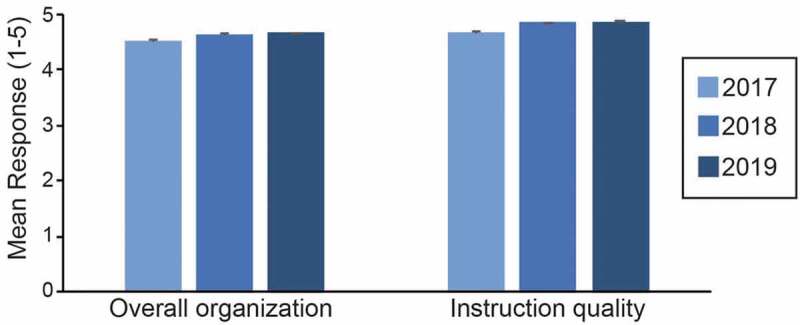
*Overall satisfaction with workshops and instructors*. Responses were collected on a five-point Likert scale: 1 = strongly disagree; 2 = disagree; 3 = neutral; 4 = agree; 5 = strongly agree. Data represent the average ratings from the answers to the questions related to overall organization and instruction quality of the workshop.

Each workshop is evaluated by participants through a survey collected at the end of each workshop. In 2017, 89% of the participants responded to the survey; 64% in 2018 and 98% in 2019. Schemes evaluating both the specifics of the workshops and their impacts on participants knowledge, have been put in place. The 2019 MU/UCB Biosciences Training Program was the most successful to date (Figure 2), reflecting a cycle of feedback and improvements after the earlier workshop series, and here we describe the strategy we used to implement a successful MU/UCB Biosciences Training Program based on this last edition.

### Call for applicants

A call for applicants was made approximately 3 months before the MU/UCB Biosciences Training Program and applicants submitted a paragraph summarizing their interest in each workshop. Each workshop was restricted to around 20 participants to keep the format interactive and suitable for in-lab learning, with preference given to graduate students and junior faculty. Applications were evaluated by a small application committee from the participating UC institutions and MU, based on several criteria including: prior training/prerequisites, demonstrated understanding of how participation in the workshop could help achieve career and academic goals, baseline understanding of scientific theory, and recommendations from faculty members. Equitable gender representation was prioritized in selecting participants, which is particularly important as being female is still considered an obstacle in pursuing a career in the sciences [15] (Table 1).

**Table 1.** MU/UCB biosciences training program participants

Year	Applied	Accepted	Male	Female
2017	NA	48	31 (64.5%)	17 (35.5%)
2018	72	71	47 (66%)	24 (34%)
2019	154	79	50 (63%)	29 (37%)

*Selected participants were PhD or MS candidates and early-stage faculty, with a select few advanced undergraduates, from the Makerere School of Biomedical Sciences, School of Public Health and School of Medicine.*

### MU/UCB Biosciences training program format

The MU/UCB Biosciences Training Program had 5 10-days workshops (Table 2), each workshop being led by an US-based faculty, supported by graduate students and postdocs from MU, UCB, UCSF and UC Davis. Morning sessions were devoted to seminar series open to all, including other students at MU. Afternoon sessions contained workshops. The seminar series ran throughout the course of the 2 weeks, with 45 min seminars led by U.S. and Ugandan faculty in the morning and afternoon, enabling bidirectional learning and the exchange of ideas and project discussions, enabling formation of intercontinental collaboration ideas. For more details on the workshops' curriculum, see supplementary data #1.

**Table 2.** The MU/UCB Biosciences Training Program workshops

Experimental Labs	
Workshops' title	Description
Protein purification	Purification of CRISPR/Cas9 for pathogen detection in complex human samples (Oren Rosenberg, MD/PhD; Cristina Tato, PhD/MPH)
Molecular cloning	Principles of molecular cloning – the art/ science of designing and assembling recombinant DNA – (Jeff Cox, PhD)
Tissue culture	Basic concepts and techniques in innate immunity (Sarah Stanley, PhD)
Non-experimental Labs	
Workshops' title	Description
Grant Writing	Introduction to Scientific Grant writing and Presentation (Nevan Krogan, PhD and Jacqueline Fabius; Michael Rape)
Bioinformatics	High Throughput Sequence Bacterial DNA Variant Analysis (Matt Settles, PhD)
Epidemiology	Application of Epidemiological Thinking and Methods to Infectious Diseases (Art Reingold, MD)

### Implementation

Separate from the curriculum, the approach to planning and implementation has been critical to the success of the MU/UCB Biosciences Training Program, to ensure sustainability and effective implementation. The involvement of local staff, students and faculty in every phase, from planning to follow-up is key for success. The efforts of the local program director were complemented by Alliance interns and the U.S. program manager. The Alliance interns were graduate students or laboratory staff from UCB, who spent 4 months leading up to the workshop learning and testing the protocols and managing communication with faculty and teaching assistants. All experiments were run several times beforehand to ensure robustness and availability of all tools and reagents. The U.S. program manager coordinated efforts and maintained communication with the local program director, serving as the U.S. liaison between MU and UCB. The U.S. program manager has traditionally been someone with experience living in East Africa or working with East African institutions, in order to maintain a level of cultural awareness. Both the U.S. program manager and the interns traveled to Kampala several weeks before the workshop, to run through protocols, review applications, and develop necessary relationships with MU faculty and staff. CEND ordered all supplies to be shipped, to be delivered to the U.S. institution initially. Supplies were repackaged to reduce shipping costs and split up into room-temperature and refrigerated shipments. This was critical for success, as direct shipping to Uganda is expensive, unreliable, and cold-chain maintenance can only be ensured if a coordinator in Uganda is proactively working with customs, expecting the shipment and ensuring dry ice or ice is refilled in case of delays. This is impossible for many small shipments but can be done for one large shipment. It required the program coordinator to be in contact with customs before the shipment was shipped, establishing a relationship with staff, and keep them notified when the shipment was expected. Like this, customs staff immediately alerted the coordinator when the shipment came in, and the coordinator travelled to the airport to personally ensure that customs were cleared without delay and transport to the university was coordinated. During the first iteration of the MU/UCB Biosciences Training Program we shipped labware, small equipment and computers that remained in Uganda, strengthening capacity for future workshops. Annual shipping includes mainly reagents for the experimental lab workshops (Figure 1). At this time, capacity exists at MU to independently handle reagent ordering, but it was more reliable and cheaper to combine all shipments in the U.S. and then closely monitor and track one large cold-controlled shipment vs. many small ones. It was surprising to note that scientists in African countries are charged higher prices for the very same reagents than scientists in the U.S. or in European countries, presumably due to the lack of local distribution networks.

To ensure workshops’ success, leadership has to be shared, not only through conception, but also during practical implementation of the program. To this end, the local program director advises on implementation and helps maintain cultural sensitivity. Both the U.S. project manager within CEND and the local program director supported the project year-round: planning for some items like, (i) pointing out grant opportunities; (ii) trying to develop collaborations and joint proposals; (iii) mentoring visiting scholars; and, (iv) supporting workshop graduates with visa application and/or travel to attend conferences. This kind of planning and interaction has to occur well in advance and follow-up is critical for long-term, sustainable success, as a continuous relationship is established and a strong connection formed between Ugandan and U.S. researchers.

## Program evaluation & outcomes

### Evaluation method

Workshop participants were asked to complete a survey immediately following the completion of the course, with questions relating to the organization of manuals/protocols, the effectiveness of instructors, the knowledge acquired, as well as overall satisfaction with the workshop. Responses were collected on a five-point Likert scale: 1 = strongly disagree; 2 = disagree; 3 = neutral; 4 = agree; 5 = strongly agree. Data represent the average ratings from the answers to the questions related to the content of the workshop. To evaluate the impact of the workshops one year after the MU/UCB Biosciences Training Program, participants were asked to fill out a one-year post-survey. In addition to surveys, impacts on participants’ skills, confidence and interest in research and their subsequent research involvement have been assessed through follow-up interviews with participants. Requests for interviews were sent to former attendees and in-person interviews with at least 10 participants were performed. While not a random selection, it is important to have a forum for student feedback independent of faculty and university leadership.

### Knowledge acquisition in a particular field

Overall, the 2019 MU/UCB Biosciences Training Program was highly rated and judged very useful: (i) 100% rating of 4 or above out of 5 for the overall satisfaction of the workshops; (ii) 97.1% rating of 4 or above out of 5 for the material used in the workshops; (iii) 98.5% rating of 4 or above out of 5 for the development of abilities and skills; (iv) 100% rating of 4 or above out of 5, for the quality of the presentation; and, (v) 100% rating of 4 or above out of 5, for the availability of the instructors.

**Figure 3.**
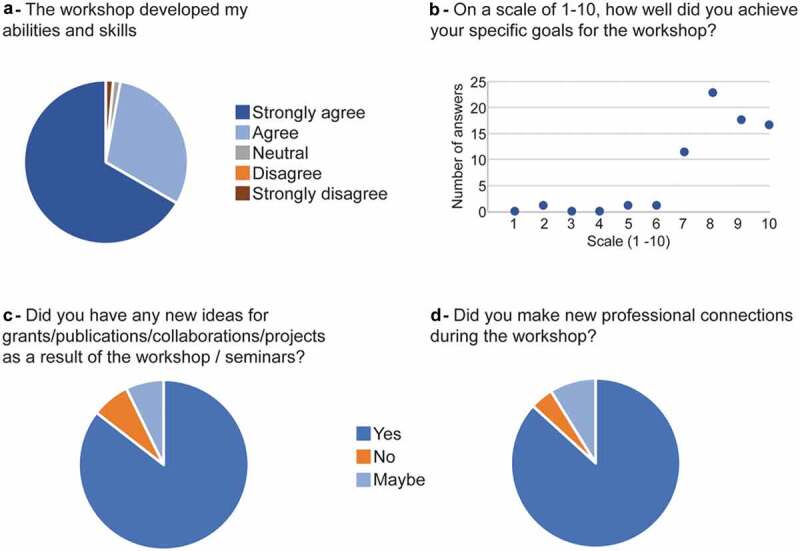
Knowledge acquisition. Mean self-reported answer to the questions concerning acquirement of knowledge of survey respondents (n = 69).

Moreover, the vast majority of students (96%) said that the workshop developed their abilities and skills and that they were able to achieve their specific goals for the workshop. 85% reported having new ideas for projects (grants, publications, and/or collaborations) as a result, and 86% reported having make new professional connections during the workshop (Figure 3 and selected participants citation below).

*“The workshop was really helpful in making me understand both literature & laboratory techniques around this study and these were because of journal clubs & practicals that were conducted.” - Participant, 2019*

*“I really wanted to learn more about experimental design as well as how to develop research proposals. I feel that I was able to learn a lot about experimental design from the practicals we went through in the lab. The journal club also gave me ideas on choosing which experiments to carry out and what to think about when you have a research question in mind.” - Participant 2019*

**Figure 4.**
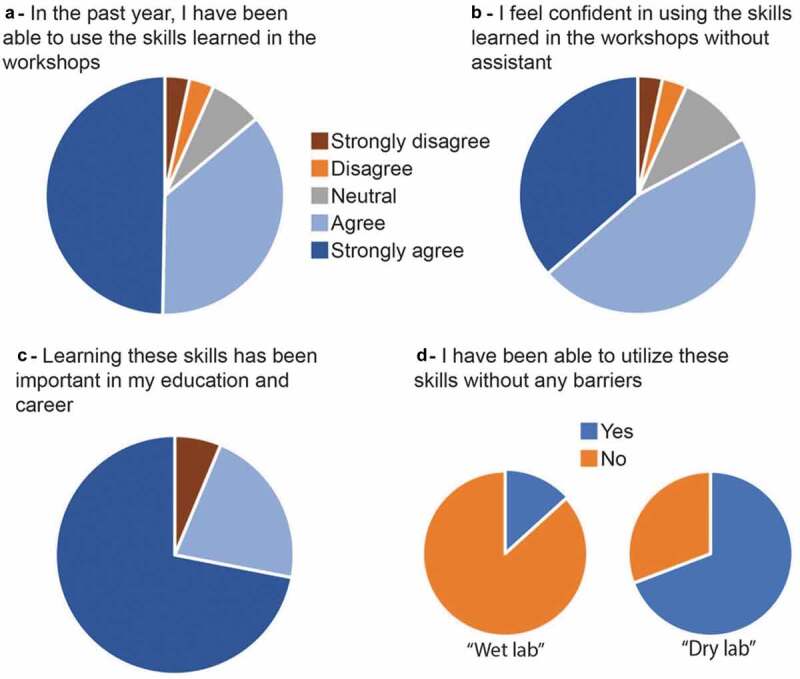
*Survey one-year post-workshop*. mean self-reported research experience after the workshops of survey respondents (50% response rate).

We also evaluated the impact of the workshops one year after the MU/UCB Biosciences Training Program. Of all the participants, 50% responded to the survey. The average ratings from the answers to the questions related to the impact of the workshops, 1 year after the MU/UCB Biosciences Training Program, are given in Figure 4. Experimental lab and non-experimental lab’s participants acknowledged that learning these skills has been important in their education and career (94% of 4 or above for non-experimental labs and 82% of 4 or above for experimental labs). However, when asked “Have you been able to utilize the skills learned without any barriers?”. 69% of non-experimental lab participants answered they were able to use their skills, in contrast of only 13% of experimental lab participants.

### Program outcomes

Our evaluation shows that, through the Alliance MU/UCB Biosciences Training Program, participants have (i) developed their abilities and skills, (ii) developed new ideas for grants, publications, collaborations and projects, and (iii) made new professional connections (Figure 3). This knowledge acquisition could lead the ground for the participants to integrate new techniques in their research, identify questions and concepts that guide scientific investigations, and design and conduct scientific research.

In particular, the grant writing workshop generated successful outcomes through scientific publications and grant applications. Several workshop participants published scientific papers [16–21], and others participated in more than ten national and international conferences abroad, such as the American Society for Tropical Medicine and Hygiene Annual Meeting. Each grant writing workshop participant wrote a grant application submitted to open external calls at the end of the workshop. Importantly, the workshop began with a joint effort of trying to identify relevant small grants through online searches as a team, which was a critical part of the learning process, as most students were not aware of all agencies and institutions providing funding to African scientists. Each student picked a small grant, most of them travel and conference grants, to work on and worked on the proposal during the workshop week, with guidance from peers and instructors. All attendees submitted a grant by the end of the week. Several were funded, which significantly boosted participants’ self-esteem and sense of accomplishment. Even participants whose grants got rejected stated that the fact that a classmate won a grant for several thousand dollars was inspiring to them and gave them confidence to seek out and pursue more grant opportunities. Strenghtening capacity by incrementally increasing self-confidence and allowing students to successfully pursue reachable goals highlights how important it is to respond to LMICs-specific needs.

Our interviews post-workshop aimed to measure the impact of the workshop on participants’ skills, confidence and interest in research and their subsequent research involvement. While hard to quantify, particularly over short time frames of evaluation, personal statements support impact on students’ confidence, performance and productivity – some examples of how students have been able to leverage the skills learned in the MU/UCB Biosciences Training Program are demonstrated:

“*I believe it [the workshop] has made me […] terrific/really good at generating great research questions/methods and choosing collaborators for my scientific research career. […]. That makes me proud.” –* Workshop participant, 2019.

*“I have designed one research project titled and given advice to so far about 7 Masters students in the Department of Immunology and Molecular Biology aiming to perform protein purification. At my lab, we have designed protein purification protocols building on the knowledge from the workshop.”* – Workshop participant, 2019.

“*[Because of my participation in the MU/UCB Biosciences Training Program] I will be co-investigator in a study which will be done with colleagues from the Medical Microbiology department that I met during the Summer workshop. We got a grant from the Makerere Research Innovation Fund using skills from the grant writing workshop*.” – Workshop participant, 2019.

As previously shown [22], this suggest that talking and learning about a new topic encourages students to explore new areas relevant to the topic. With proper guidance from experts, students feel motivated to publish their own research journals, contributing significantly to the education sector.

### Expanding participants’ networks

Of all respondents, 86% said they made new professional connections during the training program, showing that the mentorship network developed during the workshop and the seminars was helpful to students. We developed a mentoring structure allowing one-to-one explanations of key concepts, without affecting the flow of the overall workshop. To maximize learning and networking, attendance was limited to 20 participants per workshop for 4-5 teachers, including UC faculty, instructor assistants (composed of graduate students, postdoctoral fellows, and lab technicians). Moreover, the seminar series combining U.S. and African research talks allowed participants to have additional networking opportunities, present their own projects (including graduate students and postdocs), and ask specific research questions.

For example, one student got accepted as a visiting scholar into Boston University, and another into Graduate school at UC Berkeley. MU students and faculty were able to successfully fundraise and get mentoring for the First African Biomedical Scientists’ conference [23]. Communications have remained active through the rest of the year, with exchange of grant opportunities, announcements and informal mentoring of African students applying for internships or scholarshipsfor example, through feedback on application documents and through the provision of reference letters. Overall, continued mentoring support from a dedicated group of U.S. faculty during the MU/UCB Biosciences Training Program has enabled the long-term relationship between US and MU participants.

**Figure 5.**
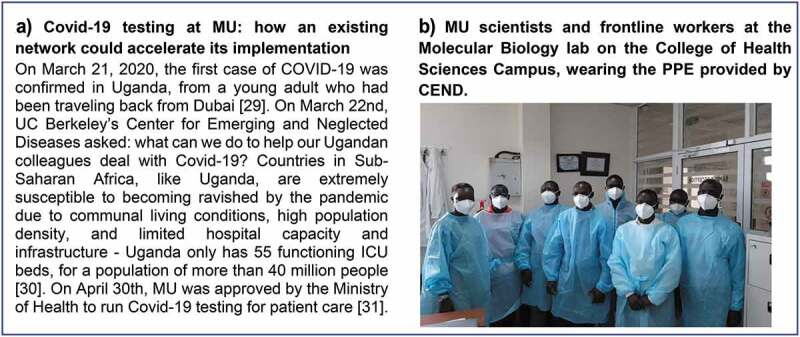
*The MU/UCB Biosciences training program generated essential collaborations*. **A) Covid-19 testing at MU: how an existing network could accelerate its implementation**. On 21 March 2020, the first case of COVID-19 was confirmed in Uganda, from a young adult who had been traveling back from Dubai [29]. On March 22nd, UC Berkeley’s Center for Emerging and Neglected Diseases asked: what can we do to help our Ugandan colleagues deal with Covid-19? Countries in Sub-Saharan Africa, like Uganda, are extremely susceptible to becoming ravished by the pandemic due to communal living conditions, high population density, and limited hospital capacity and infrastructure – Uganda only has 55 functioning ICU beds, for a population of more than 40 million people [30]. On April 30th, MU was approved by the Ministry of Health to run Covid-19 testing for patient care [31]. **B) MU scientists and frontline workers at the molecular biology lab on the college health sciences campus, wearing the PPE provided by CEND.**

The MU/UCB Biosciences Training Program also generated several collaborations, leading to successfully funded joint grant applications, such as one between a MU and UCB faculty on ‘Bacterial Factories for the Production of Diagnostic Enzymes’. An example for positive ‘ripple effects’ from capacity-strengthening efforts such as this became apparent during the early months of the COVID-19 pandemic: UCB workshop instructors had stayed in contact with MU faculty and were inquiring about the state of the Covid-19 pandemic response in Uganda, which was at the time not covered in Western news. MU feedback quickly identified a critical need around building local testing capacity and provision of PPE (Figure 5). UCB workshop instructors together with CEND and the private funders supporting the workshop brainstormed and began to raise funding. Within a week, more than $20,000 USD were raised and spent on critical needs for Ugandan researchers, testing reagents and PPE. In a highly competitive market, CEND identified a reusable face shield production company based out of Uganda and formed by a UCB graduate student, and a low-cost PPE company based out of South Africa. Working with them, this collaboration was able to secure PPE for over 200 frontline workers, who are now leading the expansive COVID-19 response in the country (Figure 5B). Using CDC-developed laboratory guidance and the protocols developed at the Innovative Genomics Institute at UCB [24], UCB research groups provided much-needed research supplies to support Covid-19 Response in Uganda and were able to get all of the lab supplies ordered and shipped out to Uganda in just a few weeks. On 30 April 2020, MU was approved by the Ministry of Health to run Covid-19 testing for patient care, and at full capacity, the molecular labs at MU are now able to run 1500 tests per day, significantly increasing in-country testing efforts (Figure 5A). This example shows how pre-existing relationships with LMIC institutions can be leveraged in times of crisis to yield benefits faster.

*‘It is encouraging to see how intercontinental collaboration between UCB and African scientists within the Alliance is leading to fast, nonbureaucratic help in times of crisis.’* UCB SPH Professor Art Reingold.

### Other benefits

The most recent MU/UCB Biosciences Training Program cost on average $2,250 per student per workshop, including accommodation and travel for U.S. participants, venue rental, and workshop supplies. During the 3 years of the program, $80,000 returned to MU including (1) $30,000 in the form of grants that were funded as a direct result of the grant writing workshop, and (2) savings of over $50,000 so far, through developing in-house methods for production of enzymes during the protein purification workshop that could be used in research projects year-round and sustainably save funds for Makerere University.

Besides the direct financial benefits generated by the MU/UCB Biosciences Training Program, the advantages to develop in country workshops go well beyond. Indeed, for a broad group of students at low cost, an in-country workshop allows (i) to support local economy through money spent locally in Kampala (lodging, food, space rent etc); (ii) all students, even the ones not participating in the workshop, to still attend talks and make connections to US researchers; (iii) better bidirectional learning opportunities; and, (iv) formation of lasting collaboration/mentorship relationships.

### Challenges & lessons learned

The main challenge to successfully implement an in-country bioscience workshop is funding, the ability to collaborate across continents and time zones, and the ability to identify reliable local partners who can be equal partners in design and implementation of the project. For the success of the 2019 MU/UCB Biosciences Training Program, both, the close communication with MU leadership and the hiring of a local program director were key. The local program director serves as the Ugandan liaison between MU and UCB and ensures that the implementation timeline (Figure 1) is followed. This helped considerably to not only be on top of any logistical challenges, but to establish a strong connection and trust with the partner university. Moreover, the ability to plan early, as well as stay creative and flexible, are key both for the workshops to be successful and for being able to overcome obstacles regarding logistics, technology, and bureaucracy. Indeed, it is important to ship early enough to allow time for delays and errors. The first year, disruptions in the cold chain rendered some reagents unusable. With enough time, we were able to reorder those reagents in the US and had faculty transport reagents personally during travel to Uganda. This allowed us to still conduct all experiments as planned, but should only be used as a last resort as transportation of biological materials on ice/dry ice is complicated and requires pre-approval procedures and compliance forms.

Another challenge faced were limitations in how many students a workshop could accommodate. Several students attempted to participate in several workshops in parallel, which was not logistically feasible due to the synchronous scheduling of the workshops. It was beneficial to discuss with faculty how many auditors could be accommodated, and having the program manager to ensure attendance was kept to the agreed upon numbers.

Curricula were also continuously improved to enable participation of all students. This was particularly valuable during the grant writing workshop, where it was found highly beneficial to focus on an individualized, interactive curriculum.

## Conclusion & recommendation

This article describes the implementation, challenges, and lessons learned of The Alliance for Global Health and Science project, aiming to develop an equity-focused model through short-term training of many scientists in-country. Many students in LMICs are underserved by current visiting scholar opportunities, and with African countries having a large population of young people, there is a big reservoir of talented students who can benefit from short-term training. By fostering knowledge and innovation in their home regions, locally-trained researchers will be better equipped to address resident health challenges.

The successful outcome of these three constitutive workshops, and the recognition that this initiative received by everyone involved, as well as long-term and unexpected benefits such as being able to work together quickly on the local COVID-19 response, encourages pursuing this approach. We believe that building personal relationships, shared ownership and trust as a foundation of the workshops and the subsequent network development, was key to the successes of the 3 consecutive MU/UCB Biosciences Training Program years.

To achieve the Alliance’s goals, we adopted a two-week format, including concurrent intensive short-term workshops taught by US faculty and their graduate students, and seminar series. This format was proved to be successful to strengthen scientific critical thinking skills and collaborative discussion.

The impacts of the Alliance were (i) acquired knowledge and practical skills necessary to pursue higher education; (ii) grants and publications; (iii) a measurable return on investment with funds returning directly to MU; and (iv) establishment of a highly skilled and well-connected community of researchers. It allows to create a network of support for each participant wanting to implement the knowledge acquired in the workshops for their own investigation and/or teaching, and a network of alumni.

## Discussion & perspectives

The limited research-related training opportunities in Africa and lack of coordinated institutional training for researchers, academic and non-academic staff, including PhD students [25] has already been described [26]. Since its inception, the annual MU/UCB Biosciences Training Program has trained more than 200 scientists and had become an essential part of the training for graduate students at MU. The success of the Annual MU/UCB Biosciences Training Program shows that workshops allow participants to develop communication skills, problem-solving skills, and other analytical skills. Participants get the opportunity to meet US faculty and students, and work with them in a workshop setting, increasing students’ networks and allowing for bidirectional learning. We think it would be worth expanding this project to other LMICs. The opportunities for African scientists to attend courses or conferences when such are organized overseas, are scarce due to the lack of external funds and often due to administrative problems, such as obtaining a visa to come to Europe or the USA. Therefore, hosting such events in various training centers in Africa would help solve these obstacles, and also serve a much larger number of students.

Instead of focusing on capacity building in the traditional sense, where the goal would be to allow MU to eventually train independently of U.S. researchers, our model allows the formation of a steady “collaborative node”, using the workshop as a training program but also as a vehicle to establish a robust, yearly forum for interaction between U.S. and Ugandan researchers, mentoring and bidirectional learning and to be able to nucleate joint ventures and collaborations. In addition, local workshops create deep, long-term mentoring relationships, formed through informal conversations, as well as long-term research partnership. Capacity is built over time as small laboratory equipment, computers, and projectors have been sent and remain at MU for future years. Currently, the yearly shipment is limited and only includes some biological reagents and writing materials such as notebooks and printed programs. We have also trained MU staff to purify certain recombinant proteins for the workshop, reducing the reliance on purchased reagents.

During the COVID-19 pandemic, workshops were adapted for online delivery, including provision of routers/internet access for all students in need. While there are challenges, both practical and methodological, in running workshops online, the workshops were well received and less costly [27,28]. We were not able to offer experimental lab workshop but we introduced two new workshops; ‘Bioentrepreneurship: Small Molecule Therapeutic and Diagnostic Development’ and ‘Scientific Diplomacy’. Although participants’ immediate evaluations of the workshops had been positive, no long-term data exists yet on the participants’ impressions of the virtual workshop. In a post-COVID world, online workshops could represent a complementary activity to the in-person workshop. We are currently working on developing a model where most laboratory-based workshops are run by MU researchers, with alumni from the last workshop leading the classes with online support from UCB faculty and students. This will allow the continuous participation of UCB faculty and students through online tools and through a dedicated website with course material that we have established for this program. UCB still helps with planning, ordering and shipping due to the lower cost. As funding allows, several UCB faculty will still travel to MU to teach and collaborate. While face-to-face interactions are preferable for networking and learning, this hybrid model significantly reduces costs of the workshop and ensures continuity. It also opens up the possibility of scaling and easier implementation at other institutions.

Altogether, this study shows that The Alliance intervention may be an equity-focused approach to expand access to research capacity strengthening in a way that empowers LMICs partners. This model for bioscience capacity strengthening in LMICs allows training of larger and diverse groups of students, development of mentoring and bi-directional research partnerships, and support of the local economy and university ecosystem by conducting training activities in Uganda. While UC Berkeley faculty have built professional partnerships with African scholars, the UC Berkeley-Alliance workshops sought to weave those often-isolated strands together, laying the groundwork for new initiatives that cross both disciplinary and geographic boundaries.

## Paper context

Science education and research have the potential to drive profound change in low- and middle-income countries (LMICs). The Alliance’s MU/UCB Biosciences Training Program, a partnership between the University of California, Berkeley (USA) and Makerere University (Uganda), is an in-country, hands-on workshop that trains a large number of students from Makerere University in molecular biology research. The current paper is a blueprint for workshop implementation, and create a valuable resource for bioscience research capacity strengthening in LMICs.
